# Examining the relationship between personality traits, anxiety levels, and coping styles with stress among architecture students

**DOI:** 10.1192/j.eurpsy.2025.1496

**Published:** 2025-08-26

**Authors:** C. Şahin, M. Karaaziz

**Affiliations:** 1 Psychiatry, University of Health Sciences Şişli Etfal Training and Research Hospita, Istanbul, Türkiye; 2 Institute of Social Science Department of Clinical Psychology, Near East University; 3 Department of Psycology, Near East University, Institute of Graduate Studies, Nicosia, Cyprus

## Abstract

**Introduction:**

The university period is a time during which individuals experience both physical and social changes, encounter conflicts and intense anxieties that can be categorized under various subheadings such as behavioral, emotional, sexual, social, economic, and academic. Moreover, studying in the department of architecture includes additional stressors that differ from other faculties, such as non-objective materials and educational outputs, the requirement for versatile abilities and originality, subjective evaluation processes, constant exposure to criticism, performance anxiety. The level of an individual’s current anxiety and the coping methods they will develop in response to stress are determined by the fundamental analysis methods of their personality traits. In this context, the aim of our study is to determine the significance of the relationship between personality traits, anxiety levels, and stress coping styles of architecture students studying at faculties located in the Marmara region, and to identify whether these factors vary according to demographic characteristics.

**Objectives:**

The levels of anxiety that arise due to various reasons, the coping styles developed in response to stressor factors, and the personality traits, which are often considered the key determinants of these two factors, are examined within the scope of architecture students, who are exposed to intense stressors as the study population. Analyzing the relationships between these elements is crucial both to address gaps in the existing literature and to contribute to public health.

**Methods:**

A study was conducted with 432 architecture students, who volunteered to participate in the study, from the Faculties of Architecture in universities located in the Marmara Region of Turkey. Data were collected using Random Sampling Method (Voluntary Basis), Online Survey Application, Socio-Demographic Information Form, Big Five Personality Inventory, Beck Anxiety Inventory, Coping with Stress Lifestyle Scale. The results of the study were evaluated using SPSS, Cronbach’s alpha analysis, Mahalanobis distance values, T-TEST, One-Way Analysis of Variance, LSD TEST, Pearson Correlation Analysis and Standard Multiple Regression Analysis.

**Results:**

As a result of the study, significant relationships were found between anxiety levels and gender, personality traits, types of schools, coping styles with stress, parental education levels; between coping styles with stress and types of schools, parental education levels, personality traits, and father’s education level, family income level; and between anxiety levels and coping styles with stress.

**Image 1:**

**Figure:**
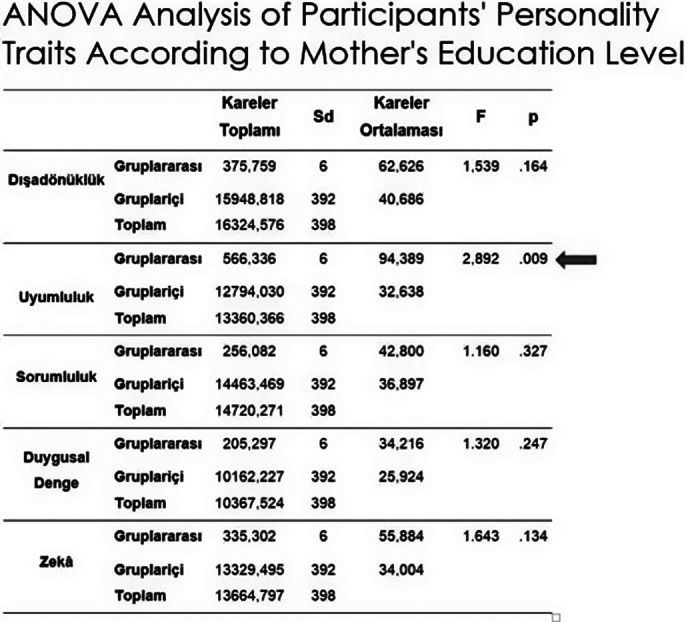

**Image 2:**

**Figure:**
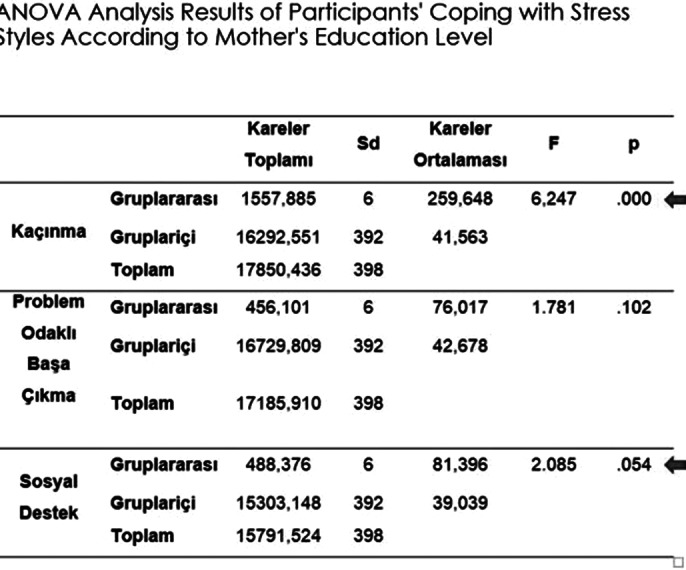

**Image 3:**

**Figure:**
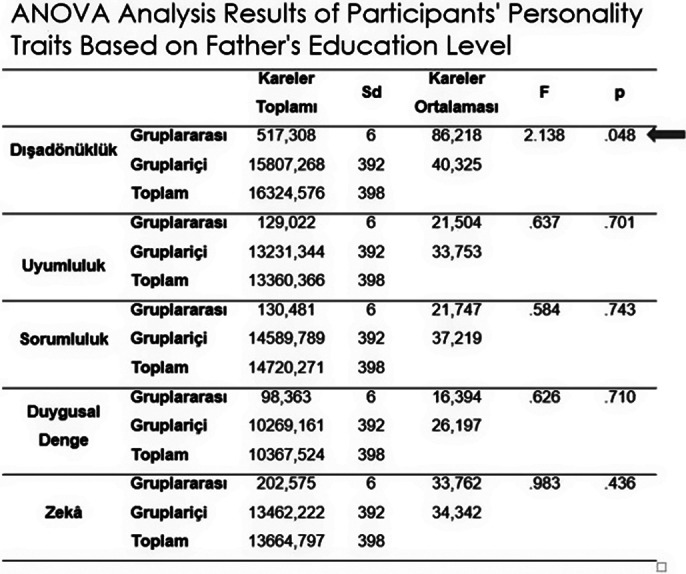

**Conclusions:**

This research has demonstrated that anxiety, personality traits, and coping styles in architecture students are influenced by various factors. This finding indicates the need for further studies focusing on architecture students.

**Disclosure of Interest:**

None Declared

